# Development of a TiO_2_/Sepiolite Photocatalyst for the Degradation of a Persistent Organic Pollutant in Aqueous Solution

**DOI:** 10.3390/nano12193313

**Published:** 2022-09-23

**Authors:** Amina Bakhtiar, Zohra Bouberka, Pascal Roussel, Christophe Volkringer, Ahmed Addad, Baghdad Ouddane, Christel Pierlot, Ulrich Maschke

**Affiliations:** 1 Unité Matériaux et Transformations (UMET), UMR 8207, Université de Lille, CNRS, INRAE, Centrale Lille, F-59000 Lille, France; 2 Laboratoire Physico-Chimie des Matériaux-Catalyse et Environnement (LPCMCE), Université des Sciences et de la Technologie d’Oran Mohamed Boudiaf (USTOMB), BP 1505, El M’naouer, Oran 31000, Algeria; 3 Unité de Catalyse et Chimie du Solide (UCCS), UMR 8181, Université de Lille, CNRS, Centrale Lille, F-59000 Lille, France; 4 Laboratoire de Spectrochimie Infrarouge et Raman (LASIR), UMR 8516, Université de Lille, F-59650 Villeneuve d’Ascq, France

**Keywords:** nanoparticles, photocatalysis, sol-gel method, sepiolite, titanium dioxyde

## Abstract

A clay-based TiO_2_ nanocomposite material was synthesized by a facile method, to investigate its structure and photocatalytic efficiency. The supported TiO_2_ nanoparticles were generated using a sol-gel method, and subsequently, mixed with a suspension of sepiolite. The material was recovered in powder form (Mc-80) and then calcined to properly arrange the crystal lattice of the TiO_2_ particles for use in heterogeneous photocatalysis (Mc-80-500). A powder X-ray diffractogram of Mc-80-500 revealed a dispersion of anatase and rutile phase TiO_2_ particles on the clay surface, exhibiting a size in the order of 4–8 nm. TEM images of Mc-80-500 confirmed the presence of isolated TiO_2_ beads on the surface of the fibrous sepiolite. The specific surface area of Mc-80-500 was larger than that of raw sepiolite and that of free TiO_2_ nanoparticles. Mc-80-500 was found to be more efficient in heterogeneous photocatalysis compared to other TiO_2_ materials based on sepiolite. Total depollution of a reactive dye (Orange G) was achieved after 1 h irradiation time, which is relatively quick compared to previous reports. The photocatalyst material can be washed with distilled water without chemical additives or calcination, and can be reused several times for photocatalysis, without loss of efficiency.

## 1. Introduction

The lack of access to safe water, sanitation, and hygiene is of deep concern to the member states of the United Nations Organisation (UNO) [1]. As part of the management of this crisis, the water post-treatment facilities of industrial plants, which discharge their effluents into the environment, need to be reviewed. Treatment of industrial discharges involves several stages (pre-treatment, clarification, primary and secondary treatment, and discharge of the effluents). However, a complementary (tertiary) treatment is often marginalized since it is considered a costly finishing phase. The final phase of an additional treatment of discharges containing non-biodegradable organic pollutants can be done using several physical, biological, or chemical methods [2,3,4]. Physical treatment is based on the transfer of material from a matrix and its storage in another phase (using an adsorbent, a membrane, etc.). The pollutant requires further treatment, as it is not yet degraded [5]. Chemical oxidation is often applied for non-biodegradable or persistent molecules. This technique is based on the activity of species with high oxidizing power such as O_3_, H_2_O_2_, O_2_, or hydroxyl radical OH°, capable of reducing or to mineralizing various harmful organic pollutants (pesticides, insecticides, nitrogen compounds, dyes, etc.) [6]. Several processes are possible such as ozonation, ultra violet (UV)/O_3_, UV/H_2_O_2_, electro-Fenton, plasma processes, and photocatalysis [6,7,8,9,10,11,12]. Photocatalysis as an additional treatment method for the purification of liquid effluents has proven to be efficient and is widely applied for the degradation of organic contaminants that have a redox potential lower than the valence band of the photocatalyst. This method represents an extremely promising alternative to conventional decontamination techniques [13,14,15,16].

The photocatalytic process is not only used to purify solutions, but can also be exploited for applications in energy and environment. Wang et al. [17] studied the design of highly efficient photocatalysts with heterostructure, for the photocatalytic splitting of water. This process (photocatalytic water-splitting (PCWS)) converts solar energy into hydrogen energy [18,19].

Various semiconductors are used for photocatalytic decontamination processes, such as SnO_2_, ZrO_2_, CdS, ZnO, CaO [16,20,21,22,23], and organic–inorganic halide perovskites [24]. However, the most widely used and effective photocatalyst is titanium dioxide (TiO_2_). For industrial applications, the photocatalysis process has to overcome two obstacles: the limit of the absorbance of the semiconductor in the ultraviolet range (wavelength corresponding to the energy required to sensitize TiO_2_ ≤ 380 nm), and the optimization of the technical, energetic, and economical costs of recovering TiO_2_ particles after photocatalysis [25,26]. The problem of improving the adsorption of the semiconductor in the wavelength range of visible light can be approached in several ways. A functionalization of the surface of the semiconductor could optimize its structure [18,19,27]. Liu et al. [18] deposited gold nanoparticles on the surface of nanorod arrays (InGaN), to obtain a photoelectrochemical water separation system. Increased photocatalytic activity occurred and was confirmed to be associated with different loading densities of gold nanoparticles. These new discoveries pave the way for scalable and economically viable solar hydrogen production. An alternative method of functionalization can be adopted; the aim is to fix the photocatalyst on a suitable support. Several reports have been published in recent years applying a wide variety of supports, such as silica gel [28]; quartz optical fibers, glass fibers, and glass beads [29]; natural and synthetic zeolites [30,31]; ceramic zeolites, cellulose fibers [32]; porous hexagonal boron nitride (BN); so-called “white graphene” [33]; graphitic carbon nitrides (g-C_3_N_4_) [24,34]; and clay [35,36]. The latter represents a natural, abundant, and low-cost material and has particularly been used as a support due to its large specific surface area [37,38], necessary for the dispersion of TiO_2_ particles [39].

**Figure 1:**
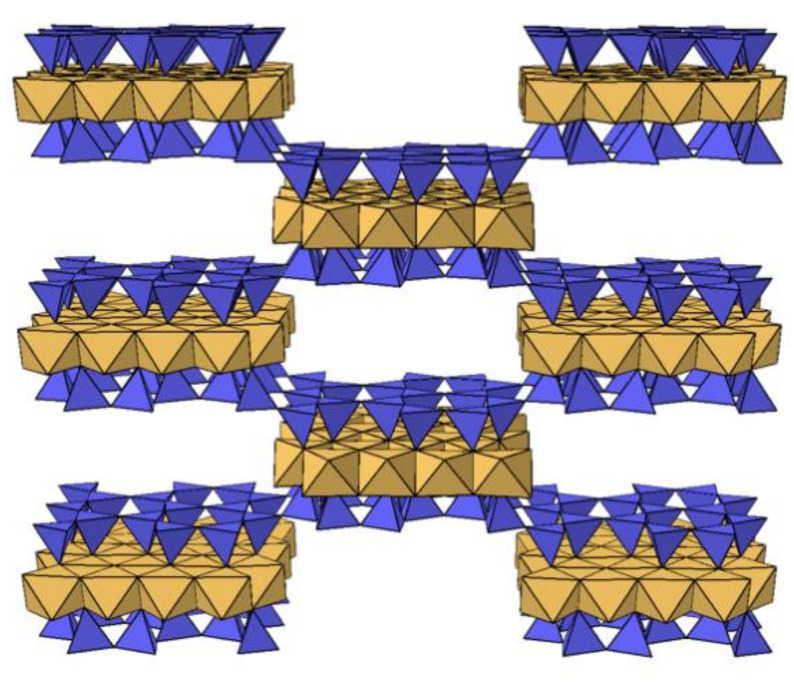
Schematic representation of the structure of sepiolite, showing Mg^2+^ as orange octahedra and Si^4+^ as blue tetrahedra. Trapped water molecules were not included for clarity reasons.

Sepiolite can be considered one of the least used clays for photocatalysis applications. Sepiolite represents a fibrous clay mineral with fine interconnected small microporous channels (≈3 × 10 Å) [40,41], having estimated pores diameter from 2 to 5 Å and usually filled by water molecules (Figure 1). Adsorption of pollutants on sepiolite has been discussed in various reports in the literature, such as the treatment of solutions containing heavy metals (such as Actinium (Ac), Pb(II), Cd(II), Cu(II), and Zn(II)) with a sepiolite bed, in order to retain the metal cations either on the surface or by trapping them in the channels [42]. Further work has been undertaken to explain the adsorption mechanism of organic pollutants such as reactive dyes on sepiolite [43]. Indeed, a good adsorption capacity of sepiolite was observed for Basic Red 46 and Direct Blue 85 dyes [44]. Only a few studies have been reported on the heterogeneous catalysis of sepiolite [44,45], related to its non-stretchable nature unlike layered silicates [46]. S. Inagaki et al. investigated sepiolite for photocatalysis applications. Desorption of water in the microporous channels by heating could crush the channels by a folding mechanism, creating structural defects that are considered the main sites of adsorption or catalytic reaction [44,47].

Ökte et al. [46] studied the structural and photocatalytic properties of TiO_2_ supported on sepiolite, prepared by a sol-gel method. The TiO_2_/sepiolite sample calcined at 500 °C showed an effect of at least partial destruction of the raw sepiolite structure. This study confirmed that the bridging of sepiolite is not easy to achieve, due to its non-stretchable nature. The specific surface area of the TiO_2_/sepiolite material (S_BET_ = 136 m^2^⋅g^−1^) was lower than that of the support (S_BET_ = 149 m^2^⋅g^−1^); probably due to the aggregation effects of TiO_2_ particles on the sepiolite surface [47]. The photodegradation of a model pollutant, β-Naphthol, was examined using TiO_2_/sepiolite material calcined at 500 °C. The photocatalysis reaction needed a long irradiation time (t_irr_ =300 min) to degrade β-Naphthol. Zhang et al. [35] developed a TiO_2_ material based on sepiolite. The results showed that the ordered structure of sepiolite in the samples heated/calcined for 2 h at different temperatures (70 °C, 200 °C, 300 °C, 400 °C) was destroyed, resulting in an exfoliated layer and other multilayered sheets. The size of the TiO_2_ particles was situated between 5 and 10 nm for the sample prepared at 70 °C. The sample calcined at 400 °C for 2 h showed a decrease of the specific surface area (S_BET_ =133.7 m^2^⋅g^−1^) compared to that of sepiolite (S_BET_ =147.5 m^2^⋅g^−1^).

Photodegradation of a model pollutant, Acid Red G (ARG), was studied using a single TiO_2_/sepiolite material heated to 70 °C [15]. The study showed that the photocatalyst took a relatively long irradiation time (t_irr_ = 120 min) to degrade a solution of ARG with a concentration of 30 mg⋅L^−1^ and with a change of pH of the solution [15].

In the present work, an in-depth study was carried out that addresses a facile and inexpensive sol-gel synthesis of a TiO_2_ material supported on sepiolite, to decontaminate aqueous solutions through a simple and efficient photocatalysis process. The developed material was analyzed using various physico-chemical characterization techniques, such as powder X-ray diffraction (PXRD), electron microscopy, porosimetry, zetametry, thermogravimetry (TGA), and inductively coupled plasma emission spectrometry (ICP-AES). These and other techniques allowed investigating the structure and properties of the synthesized TiO_2_/sepiolite material before its application as a photocatalyst. Orange G was selected as model molecule for persistent organic pollutants (POPs), to study the photocatalytic efficiency of the developed material.

## 2. Experiments

### 2.1. Chemicals

The pure sepiolite used in this study was supplied by Sigma Aldrich (Saint-Quentin Fallavier, France), characterized by XRD, SEM-EDX, and X-ray fluorescence (XRF). The chemical composition analyzed by (XRF) was found to be: 59.15% SiO_2_, 35% MgO, 2.33% Al_2_O_3_, 1.29% Fe_2_O_3_, 1.2% K_2_O, 0.35% Cl (Chlore), 0.31% CaO, and 0.18% TiO_2_ (considered as traces). The clay was used without any modifications. The following commercial reagents were used without further purification: titanium tetra chloride TiCl_4_ (Fluka, Buchs, Switzerland), purity 99.0%, absolute ethanol (Fluka) and HCl 34% analytical grade (Fluka).

### 2.2. Characterization

Several physico-chemical techniques were employed to characterize the catalyst materials. The cation exchange capacity (CEC) of the synthesized materials was calculated by the methylene blue (MB) method. Adsorption isotherms of MB/material solutions were investigated at room temperature (25 °C) and at atmospheric pressure, using a range of MB concentrations from 20 to 130 mg⋅L^−1^.

TGA was performed with a Perkin Elmer Pyris 1 analyzer (Waltham, MA, USA) with a mass resolution of 1 μg and using HT platinum plates. The analysis of the samples with an average weight of 8 mg was performed under nitrogen atmosphere, applying a flow rate of 20 mL⋅min^−1^. The samples were exposed to a heating ramp of 10 °C⋅min^−1^ in the range of temperatures between 25 and 1000 °C.

The structure and crystallinity of sepiolite and TiO_2_/sepiolite materials were analyzed by PXRD using a Siemens D-5000 diffractometer (Munich, Germany), using Cu K radiation (1.5406 Å). The ray tube was operated with a beam current of 30 mA applying a power of 40 kV. For sample analysis, 50 mg of the powder material was dispersed on a PMMA support. The rotation speed of the goniometer was 0.5°⋅s^−1^, with steps of 0.02°. The spectra were recorded for 2θ angles in a range from 5° to 80°.

Scanning electron microscopy (SEM) observations were realized using a JEOL JSM-7800F apparatus (Tokyo, Japan), operating from 0.5 to 30 kV, which was equipped with an energy dispersive spectrometer (EDX) for chemical analysis. The powder material was dispersed on a support with a double-sided conductive carbon tape. To avoid electronic charging, this preparation was completed with a nanometer-thick chrome deposit.

For transmission electron microscopy (TEM) analysis, a TECNAI-G^2^-20-Twin (Thermofischer Scientific (FEI Company), Eindhoven, Netherlands) apparatus operating at 200 kV was used, equipped with an EDX. TEM was applied to obtain images in parallel beam mode (or in scanning mode (STEM). The latter mode also allows chemical mapping of the sample with a resolution of 10 nm. TEM samples were obtained by depositing a monolayer of powder on a 3-mm diameter copper grid coated with a carbon film (transparent to electrons).

The textural properties of the prepared samples were determined using nitrogen adsorption/desorption isotherms measured with a Micromeritics ASAP 2020 analyzer (Norcross, GA, USA). The specific surface area was obtained by the Brunauer-Emmett-Teller (BET) multi-point analysis method from nitrogen or krypton adsorption at 77 K. The total pore volume was calculated from the amount of gas adsorbed at (p/p0) = 0.99, and the micropore volume was calculated using the Barrett–Joyner–Halenda (BJH) model on the desorption branch.

The chemical composition (Mg, Al, Ti, Fe, K) of Mc-80-500 and the raw sepiolite was determined with an Agilent Technologies Inductively Coupled Plasma Emission Spectrometer (ICP-AES 5110, dual view, Santa Clara, CA, USA). Acid solutions of (HF + HClO_4_/HCl + HNO_3_) were prepared to mineralize 40 mg of the ground materials, with a final volume of 10 mL in an ICP analyzer. The wavelengths and detection limit of the above elements were 279.800 nm (Mg), 396.152 nm (Al), 336.122 nm (Ti), 259.940 nm (Fe), 766.491 nm (K), and 4 µg⋅L^−1^, respectively. The spectrometer was equipped with a PTFE pneumatic nebulizer, including a double cyclonic spray chamber. The optimum instrumental conditions were plasma power: 1 kW; integration time: 5 s; background correction in dynamic mode; nebulizer pressure: 300 kPa; argon auxiliary flow rate: 1.5 L⋅min^−1^; argon plasma flow rate: 15 L·min^−1^; peristaltic pump flow rate: 15 rpm. Teflon tubes, polyethylene bottles and a pH meter (WTW) equipped with a glass electrode were also used.

Measurements of the zetapotential were made at room temperature with a Zetasizer (Nano-ZS model, Malvern Instruments, Malvern, UK). In order to study the effect of pH of the solution on the surface charge, several aqueous solutions of material/water were prepared at different pHs adjusted to given values with 0.1 N HCl or NaOH solutions. The concentration of the material was 1 g⋅L^−1^ in a volume of 10 mL. Each zetapotential value was determined in duplicate (often in triplicate) using two or three different samples.

A Cary 100 spectrophotometer from Agilent was used in reflectance diffusion mode. The Munk’s Kubelka Function was used to calculate the gap energy of photocatalyst material, in order to choose a light source that emits light of energy greater than or equal to the gap energy of the materials, in order to create the (e, hole) couple to perform a series of photodegradation experiments.

### 2.3. Preparation and Chemical Composition of Catalyst

A clay suspension (2 wt %) was stirred for 48 h, to reach saturation in distilled water; this mixture will be called mixture A. Mixture B represents a sol-gel synthesis of amorphous TiO_2_ particles: in an open reactor at 10 °C, 8 mL of TiCl_4_ was poured dropwise into 4 mL of Ethanol, with the molar ratio 1.06 (Ti/Eth) under magnetic stirring at 400 rpm. Ethanol was chosen, since a mixed anatase-rutile phase can be obtained after calcination of amorphous TiO_2_ at 500 °C [48]. Then, 4 mL of hydrochloric acid (6 N) was added dropwise to the previous solution, which acts as a peptizing agent [49] and helps to generate nanometric particle sizes [50]. The molar ratio of Ti/HCl = 0.57 was maintained. Then, 15 mL of distilled water (pH water = 5.8) was added dropwise, to hydrolyze TiCl_4_, yielding a total titanium concentration of 1.3 M. The alcohol/water ratio also controls the size of the resulting nanocrystals [51]. The sol-gel was magnetically stirred for some minutes to obtain a fresh sol-gel. The fresh sol-gel was added drop by drop to solution A, to obtain mixture C, which was left to mature for 48 h, so that the TiO_2_ particles settled on the clay fibers. Then, mixture C was washed with distilled water to remove the chlorides. Using a centrifuge, the solid part of mixture C was recovered, which was thermally pre-treated at 80 °C. A dry solid was recovered at the end of the drying process (named Mc-80), crushed with an agate mortar, and calcined in a ventilated oven. A heating ramp of 1 °C⋅min^−1^ was applied from room temperature to 500 °C, followed by an isothermal period of 12 h, before cooling the sample to room temperature with a ramp of 1 °C⋅min^−1^. The recovered calcined TiO_2_/sepiolite material is called Mc-80-500.

## 3. Results and Discussion

### 3.1. Cation Exchange Capacity (CEC)

The cation exchange capacity (CEC) was calculated for raw sepiolite, sepiolite calcined at 500 °C (sep-500), and Mc-80-500, yielding 11, 17, and 10 mg⋅g^−1^, respectively.

**Figure 2:**
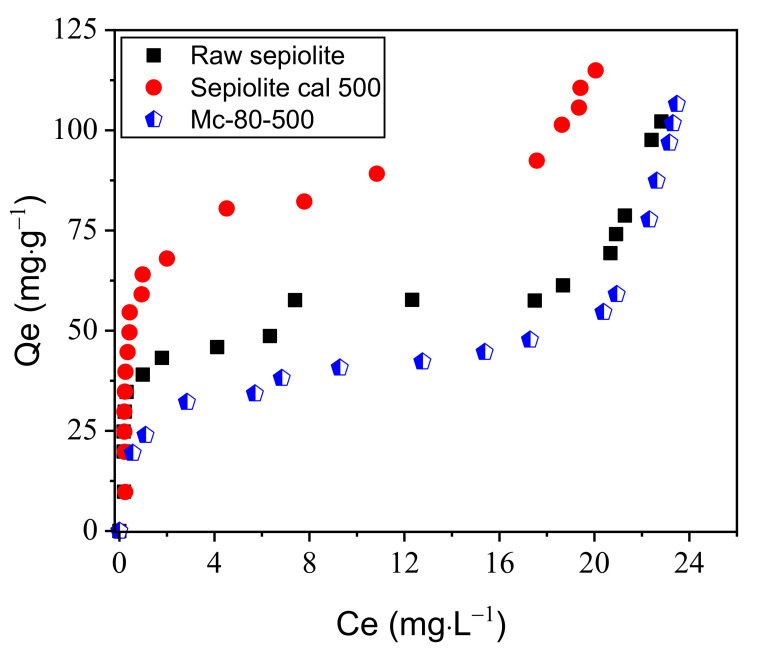
CEC of TiO_2_/sepiolite materials using the MB method.

Figure 2 presents the adsorption isotherms (Qe as a function of Ce) of the MB/material systems. Qe can be expressed as Qe = (C0 − Ce)/(V/m), where C0 represents the initial MB concentration, whereas Ce stands for the MB concentration at equilibrium. At room temperature and under atmospheric pressure, raw sepiolite is considered wet, since the channels are filled with water molecules. The cavities become free for Sep-500, i.e., new adsorption sites were created, thus explaining the difference of the maximum amount of MB adsorbed by the two clays (raw sepiolite and sep-500) [52]. Loading the raw sepiolite with TiO_2_ nanoparticles does not improve the CEC. The TiO_2_ loading is considerable for Mc-80-500, clogging the clay network and subsequently preventing cation exchange.

### 3.2. Thermogravimetric Analysis

**Figure 3:**
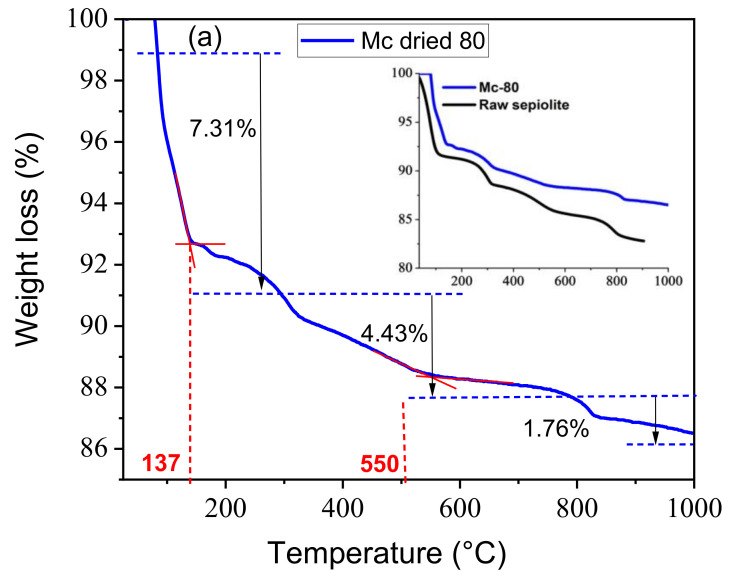

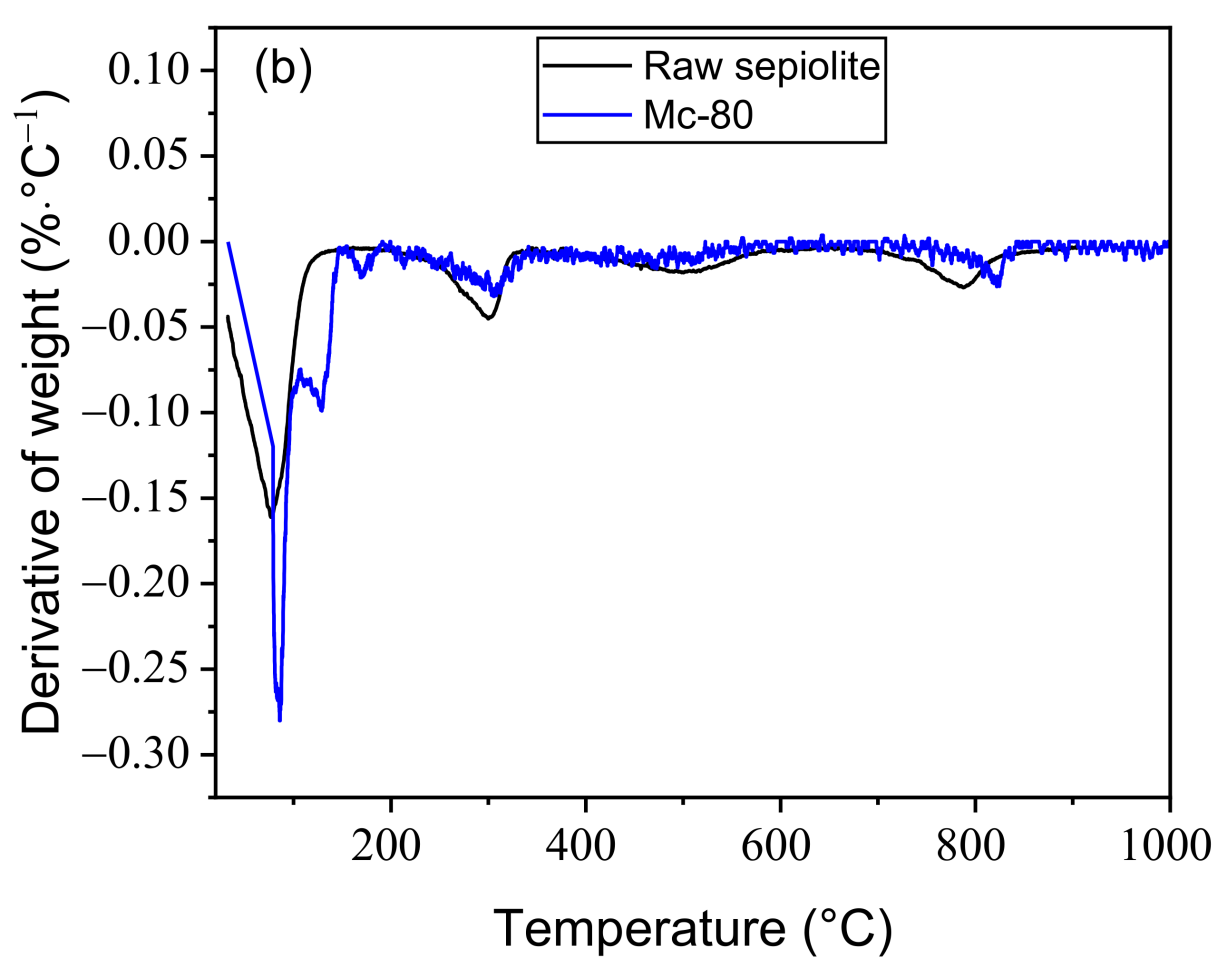
TGA results of materials, (**a**) mass loss, (**b**) mass loss derivative of raw sepiolite and Mc-80 material.

TGA plots of the raw sepiolite and Mc-80 are shown in Figure 3a,b.

Both materials showed a multi-stage dehydration process during heating in the range between 25 and 1000 °C. The analysis of raw sepiolite is consistent with the literature [53]. The first weight loss (~8%) was attributed to the loss of water molecules located in the channels. This stage ends around 130 °C and is followed by two further dehydration processes, corresponding to the departure of bound water (loss of ~3%) in the temperature range from 150 to 300 °C. Dehydration at higher temperatures (from 300 °C to 560 °C) can be attributed to the further removal of water molecules. Above 550 °C, constitutive water (hydroxyl groups associated with the octahedral sheet) is removed, and non-reversible destruction of the structure is observed. Above 700 °C, another degradation step takes place, due to the phase transformation of sepiolite to enstatite (MgSiO_3_) [46].

The following equations explain the phenomenon of heat treatment of raw sepiolite:

Step 1: Mg_8_Si_12_O_30_(OH)_4_(H_2_O)_48_H_2_O → Mg_8_Si_12_O_30_(OH)_4_(H_2_O)_4_ + 45H_2_O

Step 2: Mg_8_Si_12_O_30_(OH)_4_(H_2_O)_4_ → Mg_8_Si_12_O_30_(OH)_4_(H_2_O)_2_ + 2H_2_O

Step 3: Mg_8_Si_12_O_30_(OH)_4_(H_2_O)_2_ → Mg_8_Si_12_O_30_(OH)_4_ + 2H_2_O

Step 4: Mg_8_Si_12_O_30_(OH)_4_ → 8MgSiO_3_ + 4SiO_2_ + 2H_2_O

Interestingly, Mc-80 presents a higher thermal stability than raw sepiolite, even at high temperatures. Thus, it can be seen that the deposition of a semi-conductor (TiO_2_) enhances the thermal stability of sepiolite.

### 3.3. XRD Analysis

**Figure 4:**
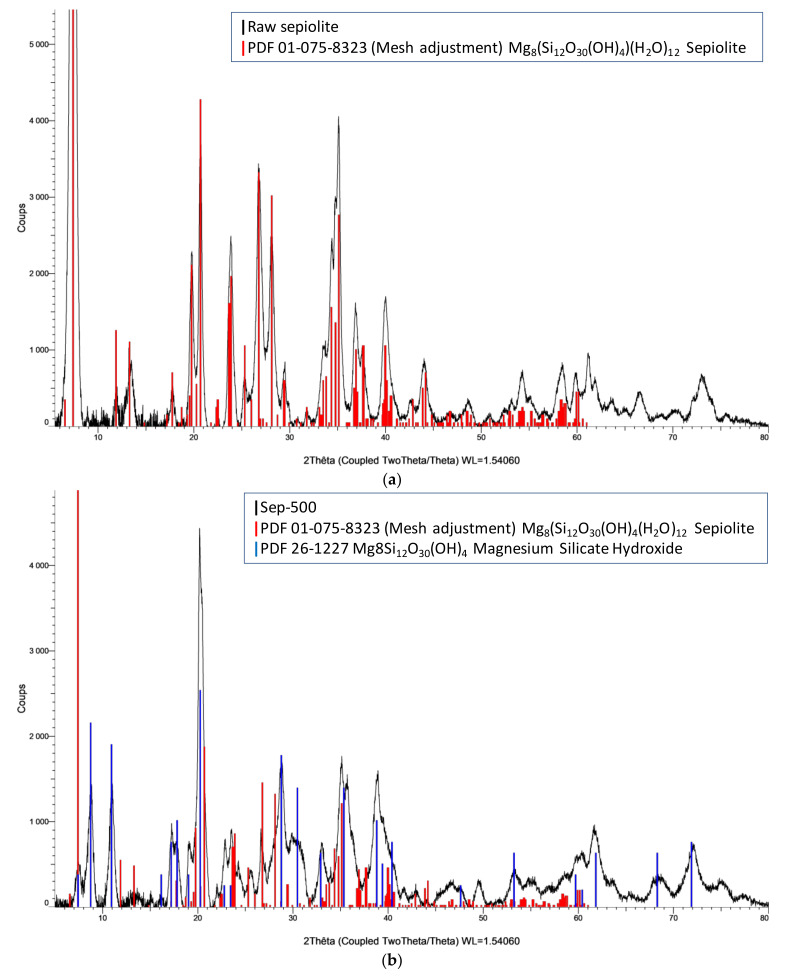

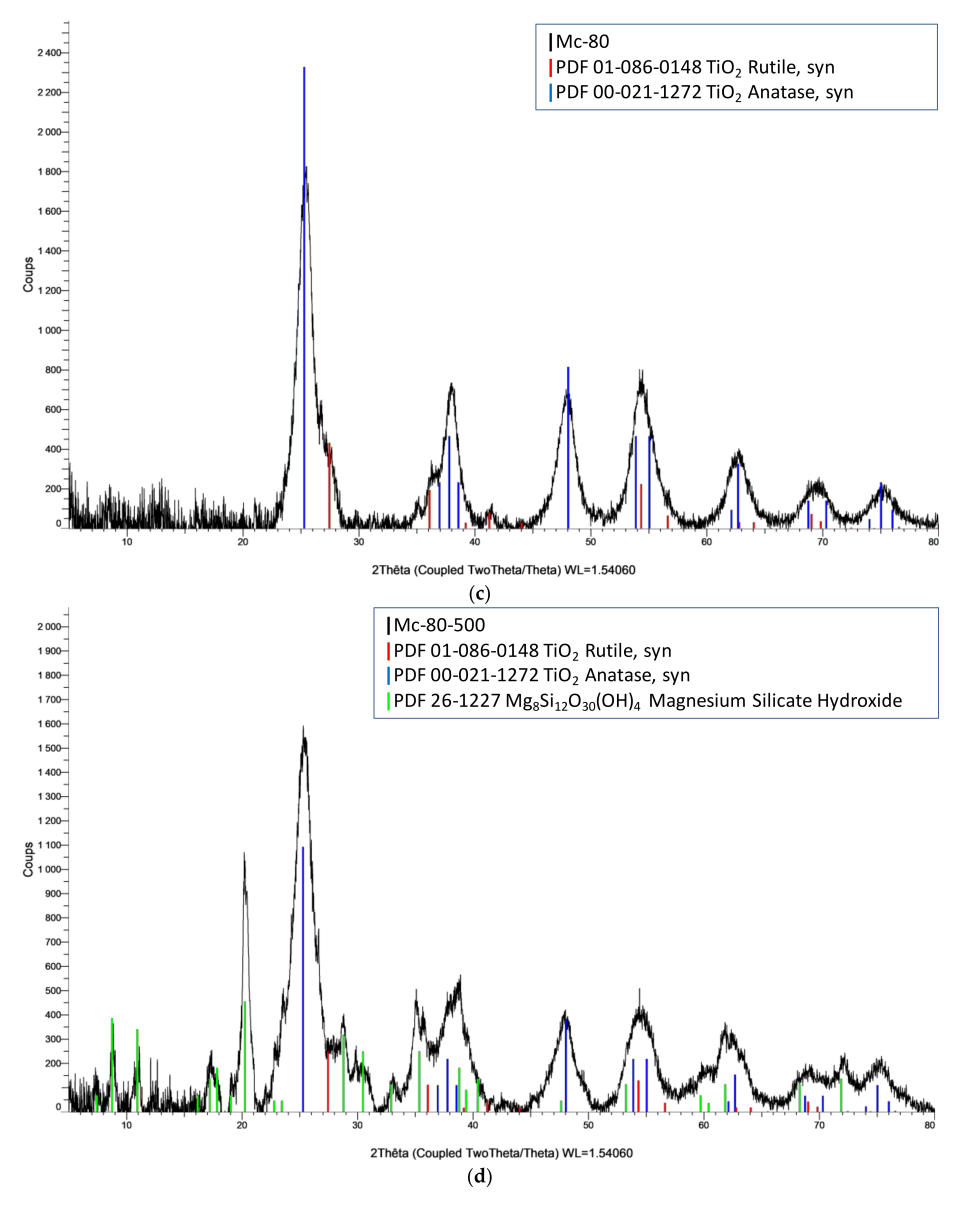

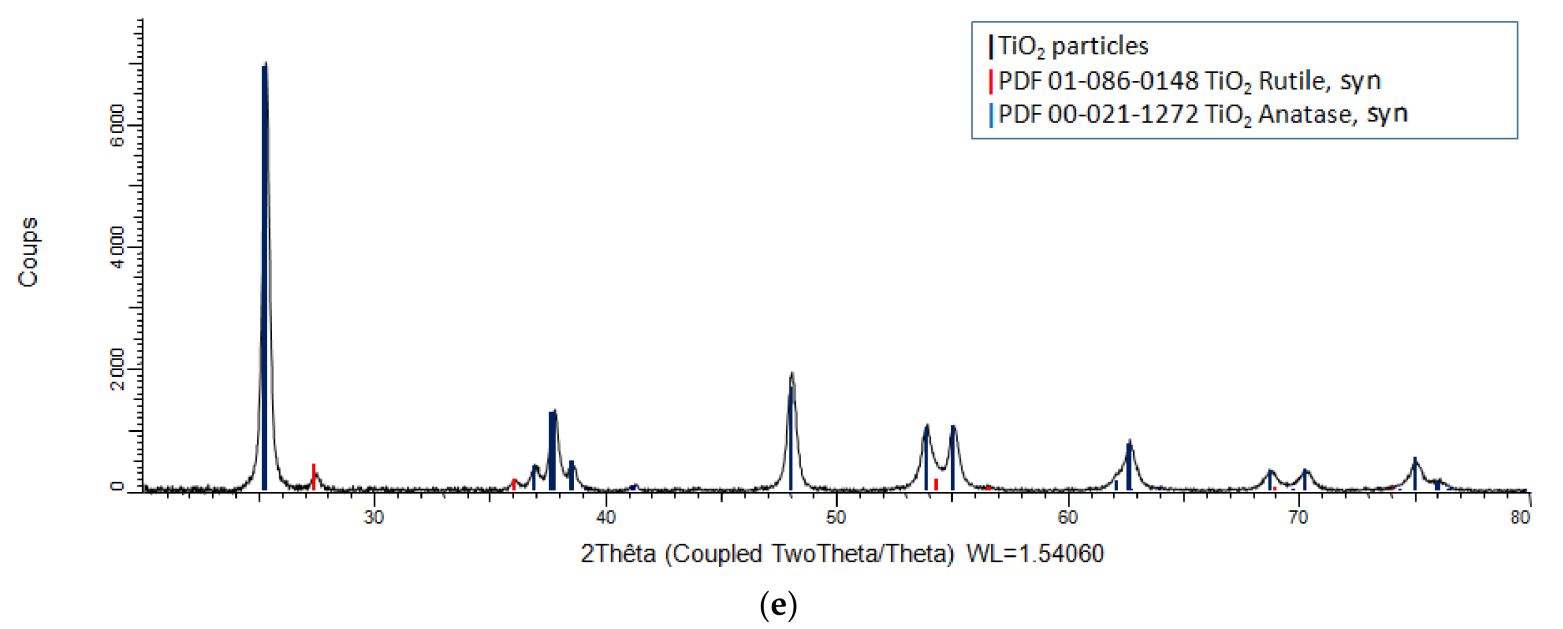
XRD diffractograms of (**a**) raw sepiolite, (**b**) Sep-500, (**c**) Mc-80, (**d**) Mc-80-500, and (**e**) TiO_2_ (without support) calcined at 500 °C.

The structure and crystallinity of the powdered materials, before (Figure 4a) and after (Figure 4b–e) heat treatment, were analyzed by PXRD. To identify the different diffraction peaks, JCPDS (Joint Committee on Powder Diffraction Standards) databases of sepiolite, partially hydrated sepiolite Mg_8_Si_12_O_30_(OH)_4_, TiO_2_ (anatase), and TiO_2_ (rutile) were used in the data processing software (Eva) (PDF 01-075-8323, PDF 26-1227, PDF 00-021-1272, and PDF 01-086-0148, respectively). The diffractogram of the raw sepiolite clearly confirms its crystalline structure (Figure 4a). Characteristic peaks of sepiolite, according to various bibliographic references, were found at 2θ = 7.44°, 12.06°, 13.38°, 17.86°, 19.90°, 20.76°, 23.72°, 26.74°, 28.12°, 35.16°, 36.84°, and 40.14°, assigned to the (110) crystallographic plane, and which correspond to the basal area of 12.16 Å, (130), (200), (150), (060), (131), (260), (400), (331), (191), (291), and (541). Indexing was carried out according to crystallographic reference sheet 26-1227, confirmed by the JCPDS sheet 75-1597 used in other similar works [54,55].

The size of the crystallites was calculated using the fundamental parameter approach over the entire powder profile using the Jana 2006 software. The software assumes that the model is isotropic and that the broadening of the diffraction peaks is due solely to the size of the crystallites. The calculated grain size of sepiolite is of the order of 11 nm. The crystalline phases present in Mc-80 and Mc-80-500 are not similar. The anatase phase (blue lines) exists in both materials, with a very small contribution of rutile (red lines in Mc-80 and Mc-80-500, Figure 4c,d). Drying of the Mc material at 80 °C (Mc-80) was able to provide sufficient energy to make TiO_2_ particles appear. A considerable amount of anatase was expressed by different characteristic peaks [35,56], whereas the peaks of anhydrous sepiolite disappeared. The sepiolite seemed to break as least partially, as mentioned in literature [35,44,47] (see also introduction of the present report). This phenomenon might be due to the incorporation of the acidic sol-gel into the sepiolite during synthesis, leading to a decrease of the reflection intensity, characteristic of the (110) plane of sepiolite [57,58].

Partial degradation of the sepiolite structure was observed for Mc-80-500, related to the acid treatment [57,59]. Pre-treatment of Mc by drying at 80 °C, and then calcining at 500 °C at a rate of 1 °C⋅min^−1^, provided sufficient energy to produce characteristic peaks of the crystal lattice of TiO_2_ that were more intense than those of Mc-80. The silica in the sepiolite was able to stabilize a large quantity of the anatase phase [60,61,62]. The size of the anatase nanoparticles for Mc-80 was found to be around 6 nm, while the size of the rutile nanoparticles was around 5 nm. The nanoparticle size of Mc-80-500 increases by 2 nm for anatase. As for rutile, it preserved its size (4 nm). It can be seen that the calcination treatment did not significantly influence the dimensions of the TiO_2_ nanoparticles. The surface presents the texture of aggregate particles. Using a silica-based matrix as support for TiO_2_ particles prevents the development of the size of TiO_2_ particles, which is beneficial for photocatalytic application [45]. The stability of the anatase phase could be related to Si-O-Ti interactions and the high dispersion of TiO_2_ particles on the surface of the silicon-rich clay matrix, thus preventing the transition of the anatase to the rutile form [63]. The diffractograms of Mc-80 and Mc-80-500, compared to the unsupported TiO_2_ diffractogram (free TiO_2_), show wider peaks. There are three causes of the broadening of XRD reflection peaks: crystallite size, lattice distortion, and lattice artefacts [61]. In the present case, after calculating the TiO_2_ particle size, the broadening of these reflections can be attributed to the small size of the crystallites deposited on the clay surface. Indeed, the size of TiO_2_ nanoparticles supported on sepiolite turned out to be much smaller than those of free TiO_2_ (without clay): anatase: 20 nm; rutile: 8 nm.

### 3.4. Microscopic Analysis

**Figure 5:**
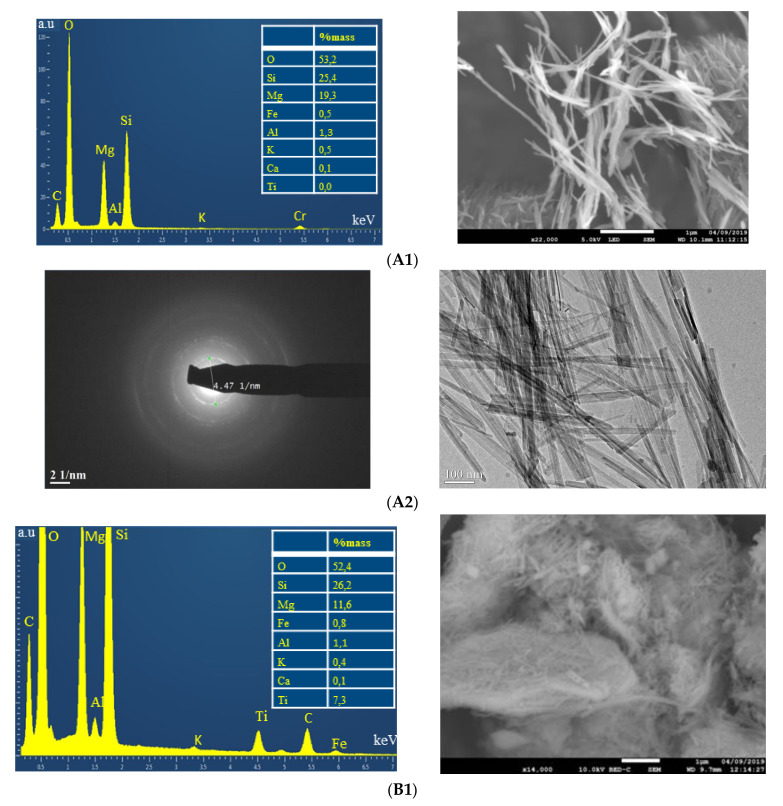

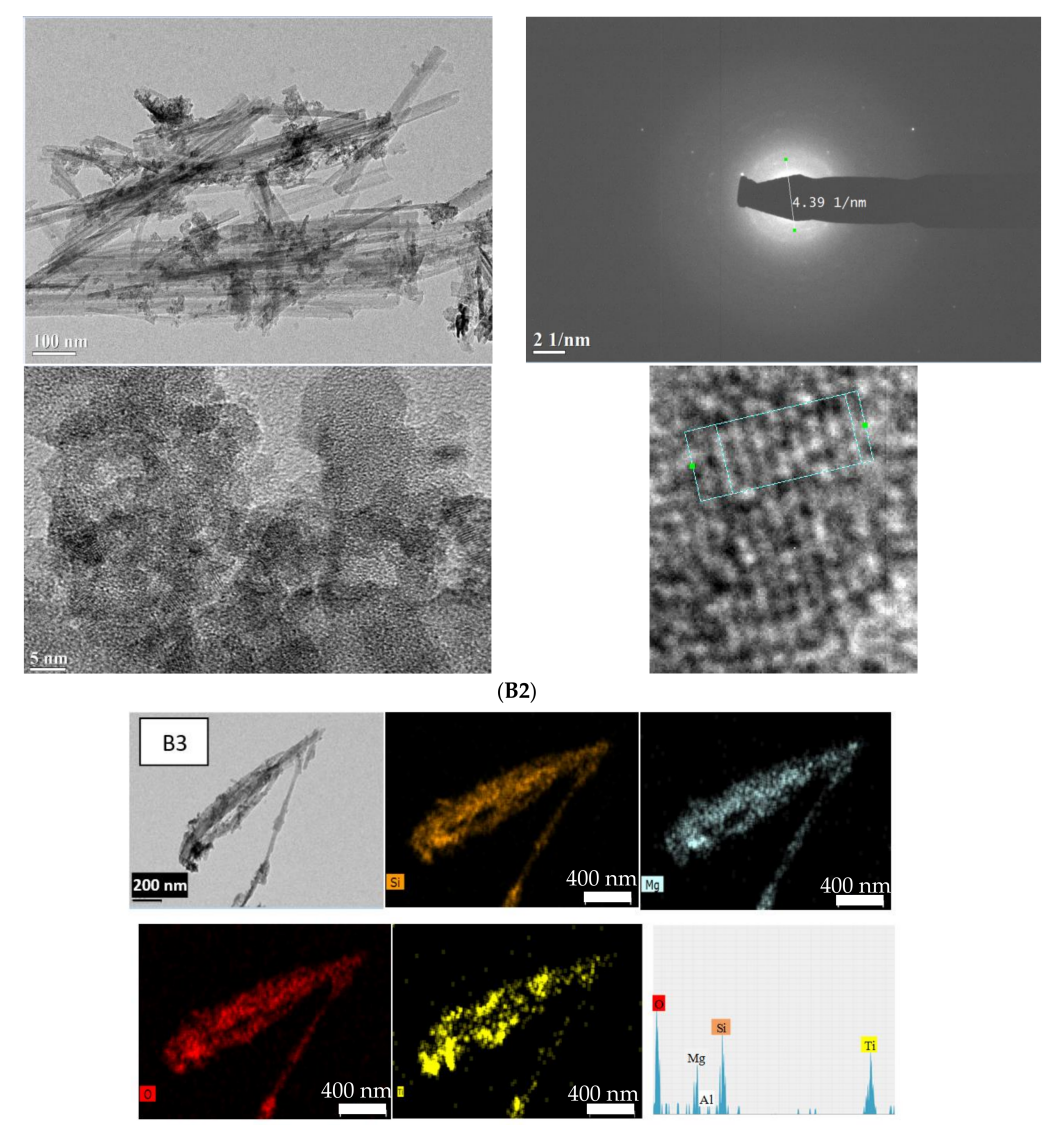
Microscopic analysis of materials, (**A1**) chemical analysis (EDX) and low magnification SEM image of Sep-500; (**A2**) TEM image of Sep-500; (**B1**) chemical analysis (EDX) and low magnification SEM image of Mc-80-500; (**B2**) TEM image of Mc-80-500; and (**B3**) chemical mapping of Mc-80-500.

The microstructure, surface texture, and chemical composition of Sep-500 and Mc-80-500 were explored via SEM and TEM analysis. Figure 5A1 represents a SEM image of Sep-500 showing a homogeneous structure, with a fiber length of several micrometres. The chemical analysis agrees well with the expected qualitative composition. The brightfield TEM images shown in Figure 5A2 confirm the SEM observations for Sep-500, with an average diameter of the fibers of 70 nm. Despite the heat treatment at 500 °C, the sepiolite retained its crystalline structure. Figure 5B1 shows a SEM image of Mc-80-500, revealing a different crystalline morphology compared to sep-500. In fact, the fibrous structure contains clusters, which turned out to be titanium rich particles. The bright field TEM images in Figure 5B2 (Mc-80-500) show that the microstructure of the sepiolite was preserved with the appearance of nanoparticles on the surface. The high resolution TEM image (the enlargement of a part of the B2 image) shows inter-reticular planes, which is in agreement with the presence of TiO_2_. The chemical mapping in Figure 5B3 confirms the presence of TiO_2_ in the sample.

### 3.5. N_2_ Adsorption/Desorption

**Figure 6:**
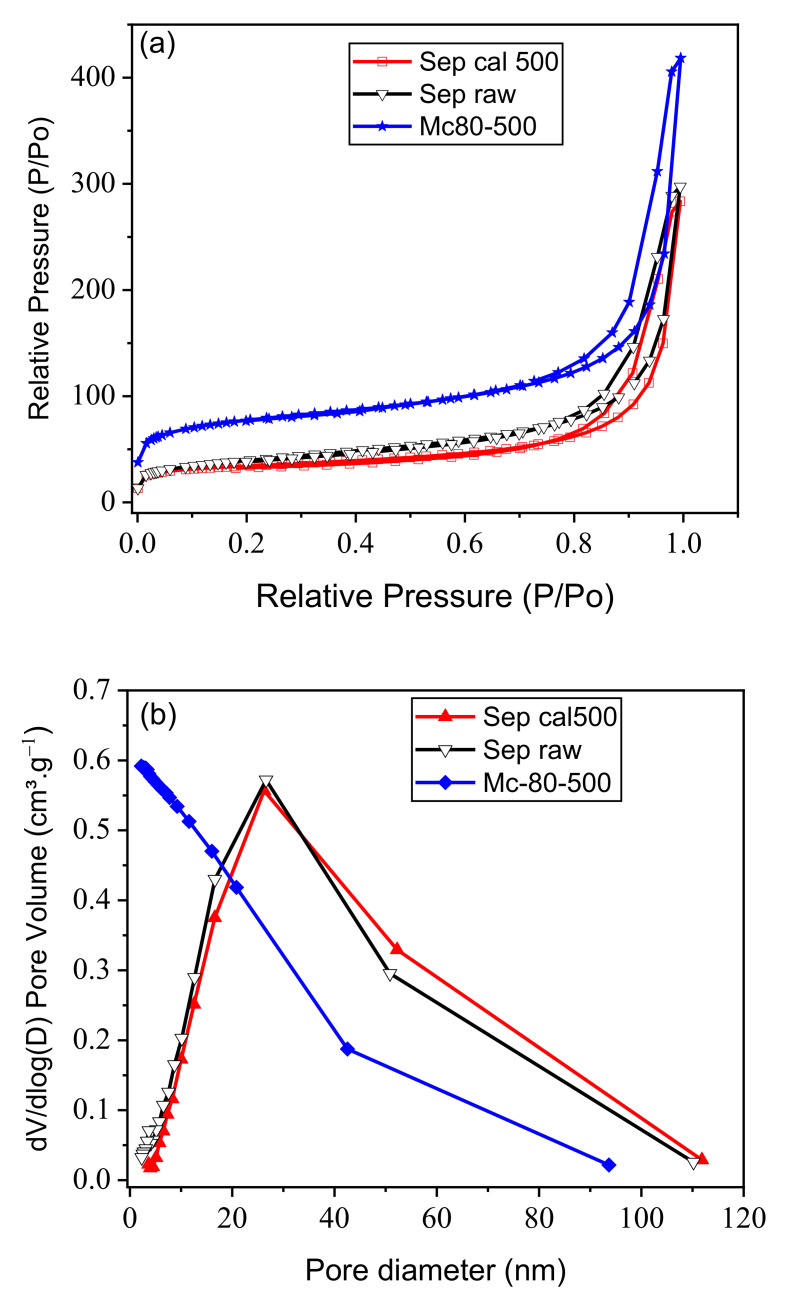
(**a**) Adsorption-desorption isotherms. (**b**) Pore distribution of the materials, raw sepiolite (black), Sep-500 (red), and Mc-80-500 (blue).

Figure 6a describes the adsorption-desorption isotherms for Mc-80-500, as well as for raw and calcined sepiolite (sep-500). All isotherms are of IV type with a hysteresis loop type H3, indicating the presence of mesopores (IUPAC). The specific surface areas were S_BET_ = 137.82, 115.68, and 261.17 m^2^⋅g^−1^ for raw sepiolite, sep-500, and Mc-80-500, respectively. The specific surface area of raw sepiolite was lower than that of other sepiolite clays: S_BET_ = 147.5 m^2^⋅g^−1^ (Hunan Province, China, Zhang et al. [35]); S_BET_ = 149 m^2^⋅g^−1^ (Anatolia, Turkey, Ökte et al. [46]); S_BET_ = 364 m^2^⋅g^−1^ (Amboseli, Kenya, Dandy et al. [52]). These differences can be attributed to the different crystallinities of these clays [47]. For the sepiolite used in this work, no impurities were detected (such as the presence of quartz) during the previous physicochemical analyses.

The pore sizes of the materials are gathered in Table 1. For Mc-80-500, TiO_2_ nanoparticles are localized on the surface of the clay, creating a kind of heteroporosity, expressed by the increase of the accessible surface. The specific surface area of Mc-80-500 is larger than S_BET_ of the raw sepiolite, indicating a good dispersion of TiO_2_ nanoparticles on the support.

The incorporation of sol-gel of high acidity, at a certain titanium concentration, caused a change of the surface of the sepiolite, since the aggressive acidity partially degrades the octahedral sheet. The residual Mg of the sheet compensates the deficit of charge in the free oxygen atoms at the edge of the tetrahedra. This breakdown explains the increase of S_BET_ of the TiO_2_/sepiolite material [59]. The sol-gel contains a mass of titanium, which is deposited on the surface, attracted by electron density maxima to compensate the deficits of the T-O-T (Octahedral sheet between two Tetrahedral sheets) skeletons [59]. Figure 6b shows the pore distribution of different materials. The pore size ranges from 2 nm to 50 nm, confirming their mesoporosity. The pore distribution curve of the clay-based TiO_2_ materials is shifted to smaller pore sizes compared to that of the raw sepiolite, which confirms the creation of a kind of heterogeneous porosity in the clay phase.

**Table 1:** (**a**) The different porosimetry parameters of materials, (**b**) comparison of porosimetry parameters of materials with those of raw sepiolite.

**(a)**						
**Material**	**S _(BET)_ (m^2^** **⋅g^−1^)**	**S_Langmuir_** **(m^2^** **⋅g^−1^)**	**Q_mBET_** **(cm^3^** **⋅g^−1^)**	**Q_m_._Langmuir_ (cm^3^** **⋅g^−1^)**	**Micropore Area (m^2^** **⋅g^−1^)**	**Vtot-Pore (cm^3^** **⋅g^−1^)**
Raw sepiolite	137	195	31	44	24	0.291
Sep-500	115	161	26	37	24	0.392
Mc-80-500	261	366	59	84	118	0.427
**(b)**						
**Material Parameters/Raw Sepiolite Parameters**					**Mc-80-500**	**Sep-500**
S _BET_ (Material/raw sep)					1.9	0.8
V _mp_ (Material/raw sep)					4.9	2.2
S _mp_ (Material/raw sep)					4.7	2.1
S_ext_ (Material/raw sep)					1.2	0.5

The ratios of the different textural parameters of the materials are summarized in Table 1b. The specific surface, microporous surface, and external surface, as well as microporous volume of Mc-80-500 present higher values compared to those of the raw sepiolite, which confirms that Mc-80-500 does not represent a simple incorporation of TiO_2_ nanoparticles, but rather a successful homogeneous deposition, expressed by the nanometric size of TiO_2_ particles on the sepiolite surface. These interpretations are in good agreement with the results of the DRX analysis of the different materials and with the corresponding SEM and TEM images [50,61,62].

### 3.6. Quantitative Inductively Coupled Plasma (ICP-AES) Analysis

The chemical compositions (Mg, Al, Ti, Fe, K) of Mc-80-500 and raw sepiolite were determined by ICP-AES. The solids were demineralized beforehand in a mixture of acidic solution. The analysis was carried out to confirm the TiO_2_ content in the composite and to calculate Mg/Ti and Al/Ti ratios, and finally to identify the main impurities. Other elements identified by ICP-AES for the samples were Na, Ca, K, and Fe. These elements are generally present in natural silicates. Silicon being a major element, its concentration does not appear in the results obtained. Mineralization of the clay by acidic attack is likely to provoke a certain volatility of silicon, so it is not possible to obtain reliable results by this method.

According to the results in Table 2, raw sepiolite contains a negligible amount of Ti. The theoretical amount of Ti incorporated in the clay phase and the actual amount analyzed by ICP-AES are distinct. This may be due to washing effects after synthesis to remove the chlorides adsorbed on the material, as well as the excess of Ti. The high acidity of the prepared sol-gel can affect the orderly structure of sepiolite from the first minutes of incorporation of the sol-gel in the clay phase. Consequently, a partial dissolution of the octahedral sheet of the structure can take place, expressed by the decrease of the concentrations of Mg^2+^, Fe^2+^, K^+^, Na^+^, and Ca^2+^ ions in the elaborated material [57,59].

### 3.7. Zeta Potential

**Figure 7:**
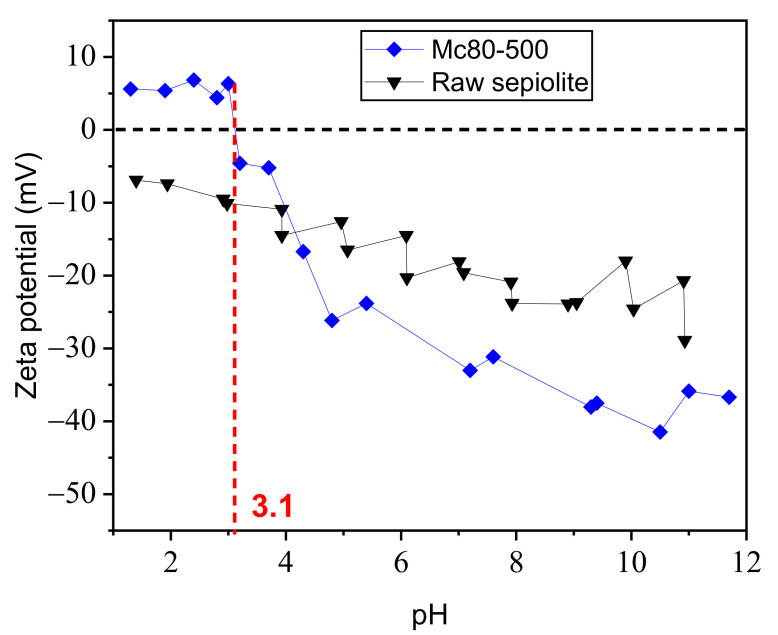
Zeta potential as a function of the pH of Mc-80-500 compared to raw sepiolite.

Several aqueous solutions of the solids (raw sepiolite and Mc-80-500) in water were prepared, with a final pH adjustment, to determine their zeta potential. The results are shown in Figure 7.

The intersection of the data with the pH axis gives rise to the point of zero charge for each material, called pH0. The zeta potential values of the studied suspensions reveal a large difference between Mc-80-500 and raw sepiolite. The latter remains negatively charged for all pH values, in the range from pH = 1.4 to 11. On the other hand, Mc-80-500 reaches pH 0 at an acidic pH of 3.1. Therefore, the modification of sepiolite by TiO_2_ deposition changes the chemical state of the surface groups of the clay.

### 3.8. Photocatalytic Efficiency of Elaborated Materials

#### 3.8.1. UV Spectroscopy of Photocatalysts

**Figure 8:**
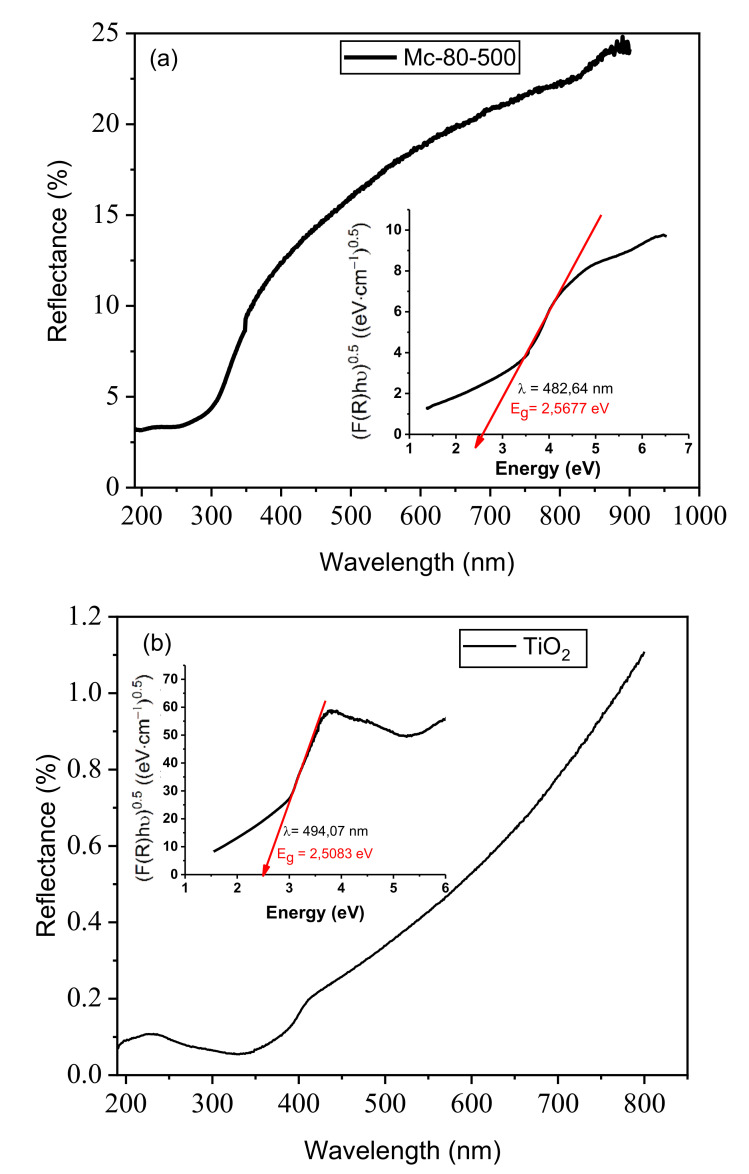
UV-visible spectra of the reflectance of materials as a function of wavelength and their corresponding gap energies. (**a**) Mc-80-500, (**b**) TiO_2_.

The following UV-visible spectrum (Figure 8) provides the values of Eg evaluated for the materials prepared. These calculations were based on plotting (F(R).h ν)^1/2^ as a function of photon energy (h ν), where F(R) is proportional to the so-called extinction coefficient, h represents the Planck constant (4.14 × 10^−15^ eV⋅s), and ν stands for the photon frequency (Hz). The resulting UV-visible diffuse reflection spectrum was obtained as a percentage of reflection R (%) as a function of wavelength.

With the reflection measurement, the Kubelka–Munk function F(R) can be calculated representing the effective absorption coefficient (α) of the sample [64].

F(R) = α= (1 − R) ^2^/2R (1)

α varies with the bandgap length of the semiconductor (Eg) and with the energy of the absorbed photon (h ν) according to the Tauc equation:

αh ν ∝ (h ν − Eg)^n^ (2)

The band gap Eg can be obtained by plotting (αh ν)^1/n^ = (F(R) E)^1/n^ against the energy h ν. It is determined by extrapolating the linear part of the energy h ν = E. The intersection of the abscissa axis (F(R) E)^1/n^ = 0 with the tangent to the linear part of the curve thus gives the experimental band gap of the sample Eg = Egraphically. The value of n depends on the type of electronic transition (n = 1/2 for the direct allowed transition).

A mass of 3 mg of solid is placed in 10 mL of distilled water under agitation, in order to analyze its absorbance and reflectance. The gap energy found for Mc-80-500 and free TiO_2_ (without support) was determined as 2.567 eV and 2.50 eV, respectively. These values correspond to the absorption wavelengths of 482 nm and 263 nm.

#### 3.8.2. Study of the Effect of pH on the Adsorption of Orange G on Photocatalysts

**Figure 9:**
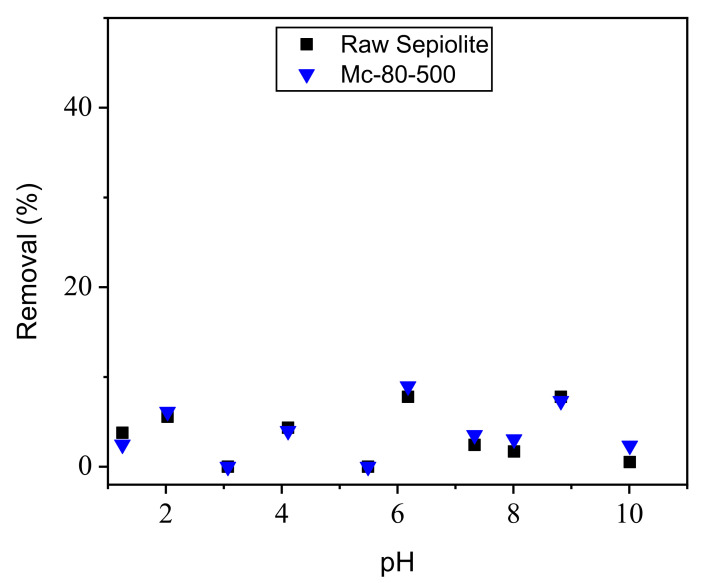
Effect of pH on OG discolorization ([material] = 1 g·L^−1^; [OG] = 30 mg·L^−1^; T = 25 °C; stirring speed = 450 tr/min; t_contact_ = 60 min).

The effect of pH on the adsorption of Orange G (OG) on photocatalysts was studied on suspensions of 30 mg·L^−1^ of OG, (solid/solution ratio = 1 g·L^−1^). The initial pH of the dye solution was adjusted to values between 1.25 and 10 by adding 0.1 M NaOH or 0.1 M HCl solutions. The suspensions were stirred for the time needed for equilibration at room temperature and then centrifuged. The dye concentration was determined by UV-visible absorption, by applying the formula Re (Removal) = (C0 − Ce)/C0 × 100%, where Re is the adsorption rate of OG by the photocatalyst, C0 represents the initial concentration of the OG solution, and Ce corresponds to the OG concentration at equilibrium. The results were presented in Figure 9, revealing only a low removal of OG by adsorption, both using raw sepiolite and Mc-80-500, independent of the pH value.

#### 3.8.3. Study of the Effect of TiO_2_ Loading in Clay on Methylene Blue Discolorization

**Figure 10:**
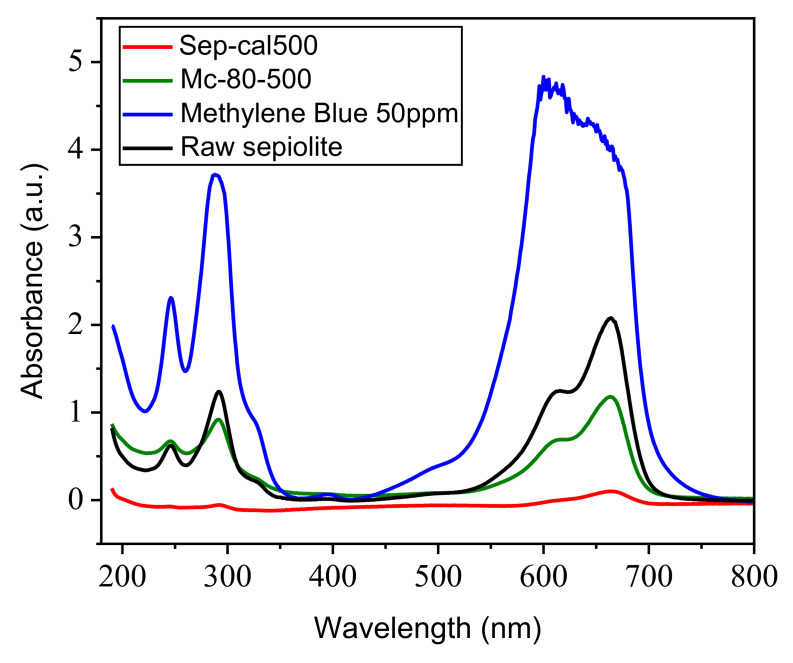
Effect of TiO_2_ loading in clay on MB discolorization ([material] = 1 g·L^−1^; [MB] = 50 mg·L^−1^; T = 25 °C; natural pH; stirring speed = 450 tr·min^−1^).

The Mc-80-500 sample was studied to examine the effect of supported TiO_2_ on the adsorption capacity of MB. According to Figure 10, sep-500, and TiO_2_/sepiolite material adsorb the reactive dye better than raw sepiolite, and this resulted from the increase of the sum of the negative charges on the surface of the clay, due to the heat treatment (for sepiolite calcined at 500 °C), and the TiO_2_ charge on the surface of the clay.

#### 3.8.4. Discolorization of Orange G with a Heterogeneous Photocatalysis Study

##### Experimental Description

**Figure 11:**
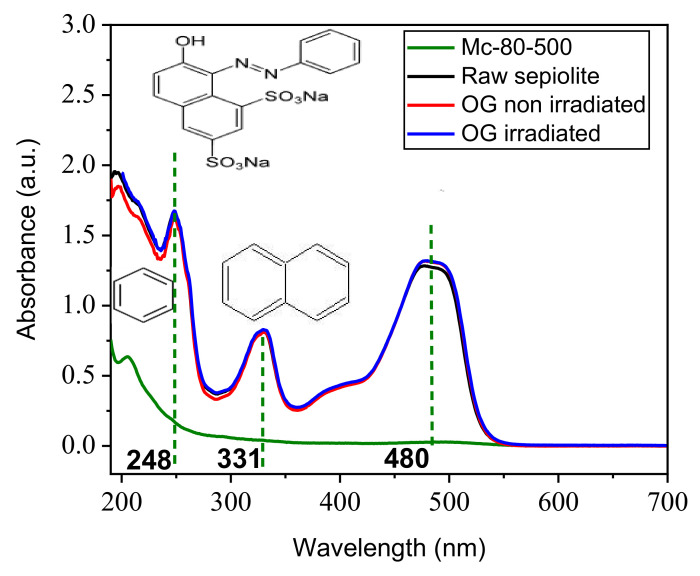
Effect of TiO_2_ loading in clay on OG discolorization ([material] = 1 g·L^−1^; [OG] = 30 mg·L^−1^; T = 25 °C; pH = 6.2; stirring speed = 450 tr·min^−1^; λ_lamp_ = 300–700 nm; t_irradiation_ = 60 min).

**Figure 12:**
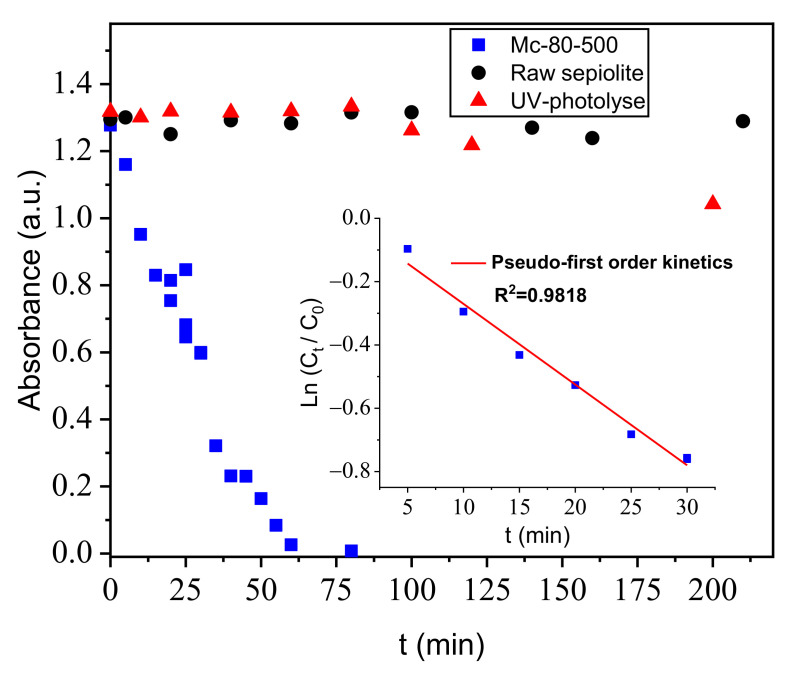
Kinetics of OG discolorization ([material] = 1 g·L^−1^, [OG] = 30 mg·L^−1^; T = 25 °C; pH = 6.2; stirring speed = 450 tr·min^−1^; λ_lamp_ = 300–700 nm; t_irradiation_ = 60 min).

Dark adsorption experiments preceded the photocatalytic experiments. The pH of (OG-material) solution was adjusted to pH = 6.2, corresponding to the value of the majority of industrial waste water containing persistent dyes. This pH value was achieved using 0.1 M NaOH and HCl solutions and a pH meter, equipped with a combined electrode. The solution remained in mechanical stirring for 1 h. The solid (mg)/pollutant (mL) ratio was 1/1, with a total solution volume (OG-Solid) of 5 mL. The photocatalytic experiments were performed in an open system, using a simple set-up at room temperature. The solution was always stirred, to maintain the homogeneity of the mixture. The light source used was a LC8 model from Hamamatsu with an intensity distribution of 20 mW/cm^2^ at 3 cm distance from the solution. Its continuous emission spectrum was located mainly in the visible wavelength range (400–700 nm), thus essentially simulating solar light. The total irradiation range was between 300 nm and 700 nm, which corresponds to an energy of 2.851 eV and 3.405 eV, respectively. At the end of irradiation, the solution was centrifuged for 5 min at 3500 rpm^−1^. Residual OG concentrations were determined using a spectrophotometer (calibration curve is C_(OG)_ = 23.183 × Abs (at λ = 478 nm) (Figure 11). In addition, all experiments were conducted at least twice. It is noted that no remarkable temperature change was detected at the time of the photocatalysis experiment. As expected; pure sepiolite had no effect on photocatalysis; it did not have a semiconductor to react with light to degrade the pollutant. The study of the kinetics of the photodegradation of OG by Mc-80-500 (Figure 12) followed the pseudo-first order model, with a correlation coefficient of R^2^ = 0.9818.

##### Comparison of the Catalytic Efficiency of the Synthesized Catalysts with Other Similar Works

The tables below (Table 3 and Table 4) gather some results from the literature, concerning photocatalytic studies of OG (or another azo dye) by TiO_2_ photocatalysts based on fibrous clay (sepiolite or palygorskite), synthesized using the sol-gel process. Table 3a indicates the irradiation time (depollution time) to degrade OG (total color removal). Much shorter depollution times to obtain total color removal were observed in this report, compared to other studies [35,58,65]. Table 3b indicates, moreover, that the catalytic efficiency of Mc-80-500 was found to be superior to that of other similar works.

Table 4, on the other hand, shows the need to perform recycling studies of the synthesized materials, in order to be able to reuse them (see Section 3.9).

##### Photocatalytic Mechanism

Several factors are likely to be the cause of the high photocatalytic efficiency of Mc-80-500, such as the size of the TiO_2_ particles, the structural properties, the charge of the surface in contact with the pollutant, and the initial pH value of the solution [55,58,66]. The nanostructured TiO_2_ particles, inserted on the sepiolite lattice, create a new system that facilitates the separation of the charges of this heterostructure, in order to favor the oxidation process and avoid the recombination between the oxide and clay charges. The textural properties of Mc-80-500, such as the increase in the specific surface are mainly due to the nanometric size of the TiO_2_ particles and their good dispersion. Since photocatalysis is a surface phenomenon, the specific surface area of Mc-80-500 plays an important role in its efficiency. One should also mention the synergistic factor between the surface of the sepiolite and the photocatalytic activity of the TiO_2_, controlled by the quantity and size of the anatase particles. During the photocatalytic process, the species formed by the action of the photocatalyst promoted the degradation process represented by the following equations [66]:

(3) $${\mathrm{TiO}}_{2}/\mathrm{Sep}+h\nu \to h_{\mathrm{vb}}^{+}+e_{\mathrm{cb}}^{-}$$

(4) $${\mathrm{OH}}^{-}+h_{\mathrm{vb}}^{+}\to {\mathrm{OH}}^{∙}$$

(5) $$H_{2}O+h_{\mathrm{vb}}^{+}\to {\mathrm{OH}}^{∙}+H^{+}$$

(6) $$O_{2}+e_{\mathrm{cb}}^{-}\to O_{2}^{-}{}^{∙}$$

(7) $$O_{2}^{-}{}^{∙}+e_{\mathrm{cb}}^{-}+{\mathrm{OH}}^{+}\to H_{2}O_{2}$$

(8) $$2O_{2}^{-}{}^{∙}+2H^{+}\to O_{2}+H_{2}O_{2}$$

(9) $$H_{2}O_{2}+e_{\mathrm{cb}}^{-}\to {\mathrm{OH}}^{-}+{\mathrm{OH}}^{∙}$$

The acid dissociation constant of OG, as well as the surface charge of sepiolite and TiO_2_, depend on the pH of the initial solution. TiO_2_ has a zero charge point = 6.2 [67], so varying the pH of the solution affects the surface charge of the TiO_2_ particles.

The surface of TiO_2_ is negatively charged in an alkaline medium (pH > 6.2), while it is positively charged in an acid medium (pH < 6.2), according to the following reactions:

(10) $$\mathrm{TiOH}+H^{+}\to {\mathrm{TiOH}}_{2}^{+}$$

(11) $$\mathrm{TiOH}+{\mathrm{OH}}^{-}\to {\mathrm{TiO}}^{-}+H_{2}O$$

where TiO^−^, TiOH, and TiOH^2+^ represent negative, neutral, and positive surface hydroxyl groups, respectively.

In this work, the photocatalytic reactions were carried out at pH = 6.2, where the adsorption of OG on Mc-80-500 is optimal (this value (6.2), close to the initial pH of the solution (5.49)). The adjustment of pH from 5.49 to 6.2 did not require a large consumption of chemical products, allowing reducing the environmental impact compared to an application of the TiO_2_/sepiolite material.

According to the literature [58], TiO_2_ has a higher oxidizing activity at acidic pH, but the excess of H^+^ type ions decreases the rate of the reaction. The photocatalytic reactions (at pH = 6.2) showed a good efficiency, this can be explained by the fact that the hydroxyl radicals (OH●) are considered as predominant species at neutral and basic pH (pH = 6–9). These radicals are easier to generate by oxidation with more hydroxyl ions available on the TiO_2_ surface, thus increasing the efficiency of the process. On the other hand, an excess of OH^−^ ions prevented the formation of hydroxyl radicals at basic pH (pH > 9). It should also be noted that in alkaline solutions, there will be a coulombic repulsion between the negatively charged surface of the photocatalyst and the hydroxide anions, which could prevent the formation of OH● and thus decrease the photo-oxidation.

**Table 3:** (**a**) Comparison of the conditions of elaboration of the material and its application in relation to similar works (TiO_2_/fibrous clay). (**b**) Comparison of the catalytic efficiency of TiO_2_/sepiolite under visible light irradiation.

**(a)**							
**Author**	**Clay**	**n Ti/g Clay (mol** **⋅g^−1^)**	**C_0pollutant_ (mg** **⋅L^−1^)**	**C_material_ (g** **⋅L^−1^)**	**Flux of Photon (mW** **⋅cm^−2^)**	**λ_irr_** **(nm)**	**Time_depollution_ (min)**
Bouna (2013) [68]	Palygorskite	0.0164	45 OG	1.5	1	365	90
Zhang et al. (2011) [35]	Sepiolite	0.03	30 ARG	1.5	20.102	253.7	90
Du et al. (2015) [65]	Sepiolite	0.0062	100 ARG	1.5	30	420	20% at 40 min
Zhou et al. (2018) [58]	Sepiolite	0.04	10 OG	0.8	300.103	365	150
This work (2022)	Sepiolite	0.004	30 OG	1	20 (distance: 3 cm)	300–700	60
**(b)**							
**Author**	**Photocatalyst**		**C_material_ (g** **⋅L^−1^)**	**C_0pollutant_** **(mg** **⋅L^−1^)**		**Catalytic Efficiency (%)**	**Time_depollution_ (min)**
Liu et al. (2017) [69]	TiO_2_/sepiolite		0.15	6 MB		58	120
Zhou et al. (2020) [70]	TiO_2_/sepiolite		0.8	10 OG		10	540
Hu et al. (2019) [71]	BiOCl/TiO_2_/sepiolite		0.6	50 simulated antibiotic wastewater (TC)		49.8	180
This work (2022)	TiO_2_/sepiolite		1	30 OG		100	60

**Table 4:** Comparison of the catalytic efficiency of TiO_2_ materials/different supports.

Author	Support Nature	Ti_loading_ (wt %)	Pollutant Nature	C_0pollutant_ (mg⋅L^−1^)	Time_depollution_ (min)	λ_irr_ (nm)	Regeneration Method
Chun et al. (2001) [28]	silica gel	30	R15 azo dye	20	40	>330	not studied
Sakthivel et al. (2002) [29]	TiO_2_ (P-25)/alumina beads TiO_2_ (P-25)/glass beads	~7–8	Acid brown 14	124	210–240	solar light	not studied
Li et al. (2008) [30]	Natural mordenite (zeolite)	5	Methyl orange	30	110	365	not studied
Trabelsi et al. (2016) [32]	TiO_2_ PC500/synthetic fibers (mainly cellulose, with some polyester)	PC500-sheet	Methyl orange	35	240	solar light	not studied
Saqib et al. (2021) [31]	Aluminosilicate (synthetic zeolite)	80	Methylene Blue	150	180	400–750	high temperature combustion and Fentonoxidation method

### 3.9. Regeneration of Functional Materials after MB Adsorption and after Orange G Photodegradation

Following the study of adsorption and heterogeneous photocatalysis, an adsorbent/photocatalyst material must undergo life cycle studies. Regeneration of the adsorbent/photocatalyst, i.e., restoration of the adsorption/photocatalysis capacity is a crucial factor for the practical application of these materials. Clays are known for their considerable adsorption capacity towards organic pollutants, either in their raw or modified forms. This opens the way to predict a relatively long material reuse cycle compared to other adsorbents, such as activated carbon with its delicate reuse operation that limits its usefulness [72].

Many studies have been carried out in order to reuse adsorbents, but the operating conditions were more or less expensive and often required the use of chemicals. In spite of all these procedures, sometimes the adsorption capacity decreases considerably after the first few cycles, even in a low-pollution environment [73]. Other methods have been used to recycle materials; i.e., after use; a suspension of TiO_2_ is mixed with the adsorbent and then exposed to irradiation, in order to clean it from adsorbates on the surface and in the cavities. This method can ensure the reuse of the adsorbent several times but it does not ensure a good cleaning efficiency. Others prefer to attach TiO_2_ particles on the surface of the adsorbent. This method generates a kind of catalyst-adsorbent with better characteristics, such as better separability or better aggregation properties. In this case, adsorption and oxidation of organic compounds occur continuously, avoiding the need for cyclic operations [58].

**Figure 13:**
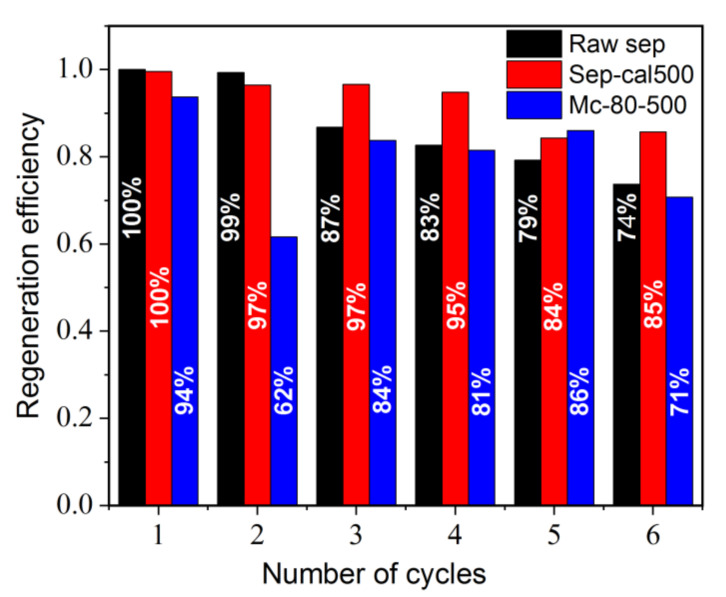
Regeneration of materials after adsorption of MB ([MB] = 20 mg·L^−1^; stirring speed 450 tr·min^−1^; t_contact_ = 60 min; λ_followed_ = 664 nm).

In order to reuse the adsorbent in an ecological way and minimize energy consumption during the recycling procedure, a simple method was adopted. The chosen pollutant was a solution of MB with a concentration of 20 mg·L^−1^ at natural pH and room temperature. This solution was adsorbed by a concentration of 1 g·L^−1^ of solid. The desorption of MB was carried out in distilled water, without the addition of other products. The washed solid was recovered by a simple centrifugation, dried, and reused for the next cycle of adsorption. The results of the regeneration are described in Figure 13. The Regeneration efficiency is expressed by the formula Regeneration efficiency = (C0 − Ce)/C0, corresponding to the MB fraction adsorbed at equilibrium. C0 represents the initial concentration of the MB solution, and Ce stands for the MB concentration at equilibrium. After five cycles of reuse, the Re of raw sepiolite and calcined sepiolite decreased to 79% and 84%, respectively. Mc-80-500 maintained its performance for MB adsorption until the sixth cycle of reuse with an efficiency rate of 70%. It is evident that the chemical nature of the solid surface (expressed in this work by the increase in the number of surface functional groups of the materials) further affects the adsorption capacity and removal mechanism of the pollutants in the solution [32,35].

**Figure 14:**
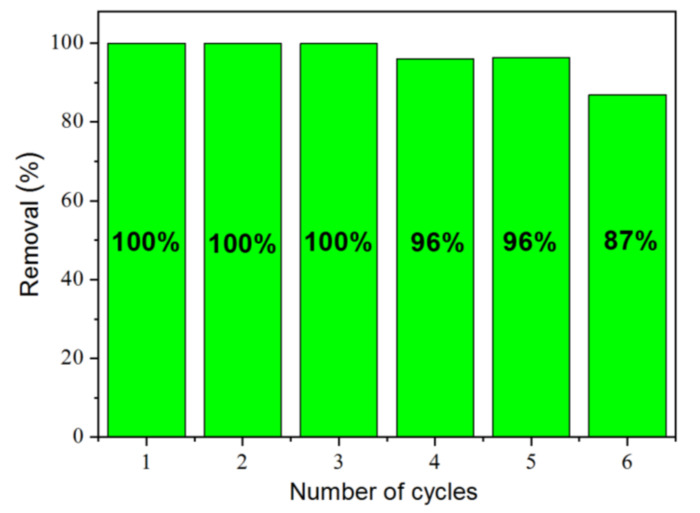
Regeneration percentage of Mc-80-500 after photocatalysis of OG ([OG] = 30 mg⋅L^−1^; stirring speed 450 tr⋅min^−1^; t_irradiation_ = 90 min; λ_followed_ = 478 nm; λ_lamp_ = 300–700 nm).

The regeneration of Mc-80-500 after photocatalysis was studied by a performing series of photocatalysis experiments using OG, in aqueous solution (Figure 14). Based on other previous works, a pH = 6 was chosen for the solution to be decontaminated, for a situation close to a real case of industrial discharge containing reactive organic contaminants.

Cycle 0 consisted of putting 5 mg of the solid in 5 mL of OG solution of concentration 30 mg⋅L^−1^ at pH = 6, mechanically stirring at 350 rpm for 60 min, to ensure adsorbate-adsorbent contact, and irradiating with a UV-visible light source (LC8) for 90 min. After centrifugation at 2500 tr⋅min^−1^ for 3 min, the supernatant was analyzed using UV-Visible absorption, following the characteristic wavelength of OG dye. The solid was then recovered, washed with distilled water, recovered again, and dried at 100 °C for 4 h to remove all excess water. The solid was ground and used for the first cycle of photocatalysis respecting the solid/solution ratio 1 g⋅L^−1^. For both studies of the reuse of TiO_2_/clay materials, MB adsorption, and OG photocatalysis, successive regenerations did not show a significative decrease in their adsorption/photocatalysis capacities. This indicates that the TiO_2_ coating on the sepiolite support was stable and did not come off during adsorption or photocatalysis regeneration processes [19,74].

## 4. Conclusions

The synthesis of a TiO_2_ nanomaterial supported on sepiolite by a facile sol-gel method at low temperature was achieved. Despite the modification of the sepiolite lattice after incorporation of the acid sol-gel (the amorphous TiO_2_ mixture), and the calcination treatment at 500 °C, the TEM images showed that the clay retained its fibrous aspect.

The developed TiO_2_/sepiolite material (Mc-80-500) contained a small amount of TiO_2_ dispersed in clay, with a higher photocatalytic efficiency compared to previous works, where sepiolite was used as a support for a relatively large amount of TiO_2_. The synthesized TiO_2_ particles had a perfect size (4–8 nm), which allowed them to be efficient in heterogeneous photocatalysis. Mc-80-500 represents a high-performance material; it can be effective in the visible irradiation range and recycled at least six times, with high efficiency, to transform a persistent molecule (Orange G dye) into benzene molecules. The irradiation time to degrade Orange G was much shorter compared to other studies [35,58,65].

The recycling of the elaborated sepiolite material is rather simple, it consists in a washing procedure with water without adding chemicals, and drying to return it to its initial state (reusable powder material).

In general, the synthesis of TiO_2_/sepiolite material is not expensive; the TiO_2_ nanoparticles are well immobilized on the clay. It is very efficient in photocatalysis and recyclable.

## Figures and Tables

**Table 2:** ICP-AES analysis of some chemical elements in a 40 mg mass of material.

Concentration (mg⋅g^−1^)						Ratio	
	Al	Ti	Mg	Fe	K	Ti/Mg	Ti/Al
Raw sepiolite	2.29	0.61	13.79	2.86	7.88	0.04	0.26
Mc-80-500	3.78	191	0.93	0.44	4.29	205.4	50.53

## Data Availability

Data are contained within the article.
